# Usefulness of SS18-SSX antibody as a diagnostic marker for pulmonary metastatic synovial sarcoma

**DOI:** 10.1186/s13000-021-01110-6

**Published:** 2021-06-14

**Authors:** Kentaro Miura, Kimihiro Shimizu, Takashi Eguchi, Sachie Koike, Shunichiro Matsuoka, Tetsu Takeda, Kazutoshi Hamanaka, Takeshi Uehara

**Affiliations:** 1 Division of General Thoracic Surgery, Department of Surgery, Shinshu University School of Medicine, Matsumoto, Japan; 2 Department of Laboratory Medicine, Shinshu University School of Medicine, Matsumoto, Japan

**Keywords:** Synovial sarcoma, SS18-SSX antibody, Immunohistochemistry, Pulmonary metastasis

## Abstract

**Background:**

The novel SS18-SSX fusion-specific antibody is reported to have high sensitivity and specificity for the diagnosis of primary synovial sarcoma (SS), which often metastasizes to the lung. Thus far, no study has validated the diagnostic efficacy of SS18-SSX antibody for pulmonary metastatic SS. Therefore, we aimed to investigate the usefulness of the SS18-SSX antibody in the diagnosis of pulmonary metastatic SS.

**Methods:**

We evaluated the immunohistochemistry of SS18-SSX fusion-specific antibody (E9X9V) in 10 pulmonary metastatic SS cases and the corresponding five primary sites (four limbs and one mediastinum) in five patients, for whom SS was already diagnosed and confirmed by fluorescence in-situ hybridization in the metastatic and primary sites, and in 93 clinical and histologic mimics including 49 non-SS, pulmonary metastatic sarcomas, 39 primary lung cancers, and five intrathoracic solitary fibrotic tumors. All specimens were surgically resected at Shinshu University Hospital during 2001–2019. For primary and metastatic SS, we also evaluated SS18-SSX immunohistochemistry using needle biopsy and touch imprint cytology specimens from the primary site.

**Results:**

SS18-SSX staining was diffusely-strongly positive in all 10 pulmonary metastatic SS cases and the corresponding five primary sites; whereas, it was negative in all 93 clinical and histologic mimics (100% sensitivity and 100% specificity). Further, SS18-SSX staining was also sufficiently positive in the biopsy and cytology specimens.

**Conclusions:**

Immunohistochemistry of the SS18-SSX fusion-specific antibody is useful for the differential diagnosis of pulmonary metastatic SS in clinical practice. This simple and reliable method has the potential to replace traditional genomic tests. However, further studies are warranted in this regard.

## Background

Synovial sarcoma (SS) is a malignant mesenchymal neoplasm with varying epithelial differentiation, and accounts for 5–10% of all soft tissue sarcomas [1–4]. SS frequently occurs in the limbs and occasionally in the chest cavity. Approximately 50% of the patients with SS experience metastasis, which is commonly observed in the lungs and pleura [3, 5]. In patients with resectable pulmonary metastases of SS, the mainstay of treatment is pulmonary metastasectomy, which is reported to show good prognosis [6–8]. Several studies have reported that repeated pulmonary metastasectomies would be a feasible strategy for select patients with metastatic SS [9, 10]. Therefore, thoracic surgeons frequently encounter cases of pulmonary metastatic SS in clinical practice.

A diagnosis of SS is made depending on the presence of the hallmark t(X;18)(p11;q11) translocation, which is not found in other neoplasms [4, 11]. This recurrent translocation results in the fusion of the SS18 gene on chromosome 18 with one of the several SSX genes on chromosome X (SSX1 in two-thirds of SS, SSX2 in one-third, and SSX4 rarely), which create SS18-SSX fusion oncogenes in > 95% of the cases [4, 11, 12]. Histologically, SS is divided into two major subtypes (biphasic type and monophasic spindle cell type) and other rarer subtypes (monophasic epithelial, poorly differentiated, calcifying/ossifying, and myxoid types) [3, 4]. Owing to the varying histological combinations of epithelioid and spindle cells, the differential diagnosis of SS widely ranges from non-SS bone and soft tissue sarcomas to various types of carcinomas. Therefore, making a definitive diagnosis of SS based only on histological findings is difficult, and genetic confirmation of the SS18-SSX fusion by fluorescence in situ hybridization (FISH) or reverse transcriptase-polymerase chain reaction (RT-PCR) has been the gold standard for the diagnosis of SS [3, 4]. However, these tests are not widely available because of their high cost and time-consuming process [13].

In patients with pulmonary metastatic SS, clinical course, radiologic features, and histologic findings are widely variable, resulting in difficulties in the differential diagnosis of SS from clinical and histologic mimics such as primary lung cancer and other bone/soft tissue sarcomas [3, 14, 15]. Particularly in cases of late and solitary pulmonary metastatic SS, the differential diagnosis would be difficult but clinically important. Therefore, a clinically useful and pathologically accurate test for the diagnosis of metastatic SS is warranted.

Recently, Baranov et al. proposed a novel diagnostic test for primary SS using immunohistochemistry (IHC) of the SSX-SS18 fusion-specific antibody, with high sensitivity (95%) and specificity (100%) [4]. However, it has not been clarified whether SS18-SSX IHC can be used as a marker to diagnose pulmonary metastatic SS, particularly during differential diagnosis from clinical and histologic mimics such as primary lung cancers and pulmonary metastatic non-SS sarcomas.

The present study aimed to evaluate the usefulness of SS18-SSX IHC in the diagnosis of pulmonary metastatic SS and the diagnosis of the rare mediastinum-originated SS and the potential utility of biopsy and/or cytology specimens for the IHC test.

## Methods

### Study cohort and design

We retrieved the details of patients with SS, who underwent surgical resection between 2001 and 2019, from the Pathology Department of Shinshu University Hospital. The whole slides of the tumor of five patients were available for 10 pulmonary metastatic SS and the corresponding five primary sites (four in the limbs and one mediastinum), and of 93 clinical and histologic mimics, including 49 pulmonary metastatic bone and soft tissue sarcomas other than SS (osteosarcoma, chondrosarcoma, liposarcoma, malignant fibrous histiocytoma, Ewing’s sarcoma, leiomyosarcoma, fibrosarcoma, and undifferentiated pleomorphic sarcoma), 39 primary lung cancers (adenocarcinoma, squamous cell carcinoma, small cell lung cancer, large cell lung cancer, pleomorphic cell carcinoma, and carcinoid), and 5 intrathoracic solitary fibrotic tumors. All patients with SS underwent surgical resection of the primary origin and were diagnosed with SS by SS18 break-apart FISH to detect the SS18-SSX fusion gene. In metastatic SS specimens, two metastatic sites were selected from each patient, and a total of 10 specimens were stained. Chemotherapy had been initiated for all patients before the resection of the metastatic specimens. SS and other bone and soft tissue sarcomas were consecutive cases, including repeated pulmonary metastasectomies. Patients with SFT and primary lung cancer were randomly selected from our database. This study was approved by the Shinshu University Research Ethics Committee (No. 4870).

### Immunohistochemical staining

IHC staining was performed by manual methods. Specimens of whole tumors (SS, other bone soft tissue sarcoma, primary lung cancer, and solitary fibrotic tumor [SFT]) were paraffin-embedded and cut into 4-μm-thick sections. They were deparaffinized with ethanol and xylene, and endogenous peroxidase activity was blocked using methanol and 30% H_2_O_2_ solution for 30 min at room temperature. Protein blocking was performed using 1% bovine serum albumin [BSA]/phosphate-buffered saline [PBS] for 1 h at room temperature. The sections were incubated overnight at 4 °C with primary antibody against human SS18-SSX (clone E9X9V, 1:1000, Cell Signaling Technology, Danvers, MA, USA). The sections were washed in PBS three times and probed with an anti-rabbit IgG labeled with Histofine Simple Stain MAX-PO (Nichirei, Tokyo, Japan) for 1 h at room temperature. They were washed three times in PBS, and the immune complex was visualized using Histofine Simple Stain 3,3′-diaminobenzidine (Nichirei, Tokyo, Japan). After washing in water, the sections were counterstained with hematoxylin.

Immunostaining of intraoperative sealed cytology was performed similarly. The tumor was directly smeared onto the slide grass and then fixed with 99.5% ethanol. The subsequent immunostaining steps were the same.

Immunoactivity was reviewed by one pathologist (UT) and one thoracic surgeon (MK).

## Results

### Characteristics of patients with synovial sarcoma

The characteristics of patients with SS are presented in Table 1. All SS of primary origin and pulmonary metastasis had already been diagnosed using SS18 break-apart FISH. The age at the identification of the primary tumor as SS was considered for each patient. In all five patients with SS, at least two metachronous metastasectomies were performed. The duration between the resection of primary sites and pulmonary metastasectomies ranged from 28 to 108 months. Cases 1, 3, 4, and 5 were monophasic spindle cell types, and Case 2 was biphasic type.

**Table 1:** Characteristics of the five patients with synovial sarcoma who underwent pulmonary metastasectomy

Case no.	Age, years	Sex	Primary	State	Type	1st	2nd	3rd	4th	5th
**1**	63	F	Ankle	Dead	Monophasic	Left wedge resection (1)	Right wedge resection (2)	Left wedge resection (1)		
						39 months	59 months	61 months		
**2**	28	M	Mediastinum	Alive	Biphasic	Right lower lobectomy (1)	Left wedge resection + chest wall resection			
						108 months	177 months			
**3**	33	M	Forearm	Alive	Monophasic	Right basal segmentectomy (1)	Left wedge resection (1)	Left basal segmentectomy (1)	Left wedge resection (1)	
						48 months	63 months	82 months	100 months	
**4**	51	M	Knee	Alive	Monophasic	Right wedge resection (2)	Left wedge resection (2)	Right wedge resection (1)	Left wedge resection (1)	Right wedge resection (1)
						28 months	59 months	76 months	97 months	108 months
**5**	30	M	Forearm	Alive	Monophasic	Right wedge resection (3)	Left wedge resection (2)	Right wedge resection (2)	Left wedge resection (3)	
						32 months	34 months	68 months	107 months	

The age at which the primary tumor was identified as SS was considered for each patient. The months listed below the operative procedure indicates the duration since the identification of the primary origin. The number in parentheses next to the surgical procedure indicates the number of resected tumors

**Fig. 1:**
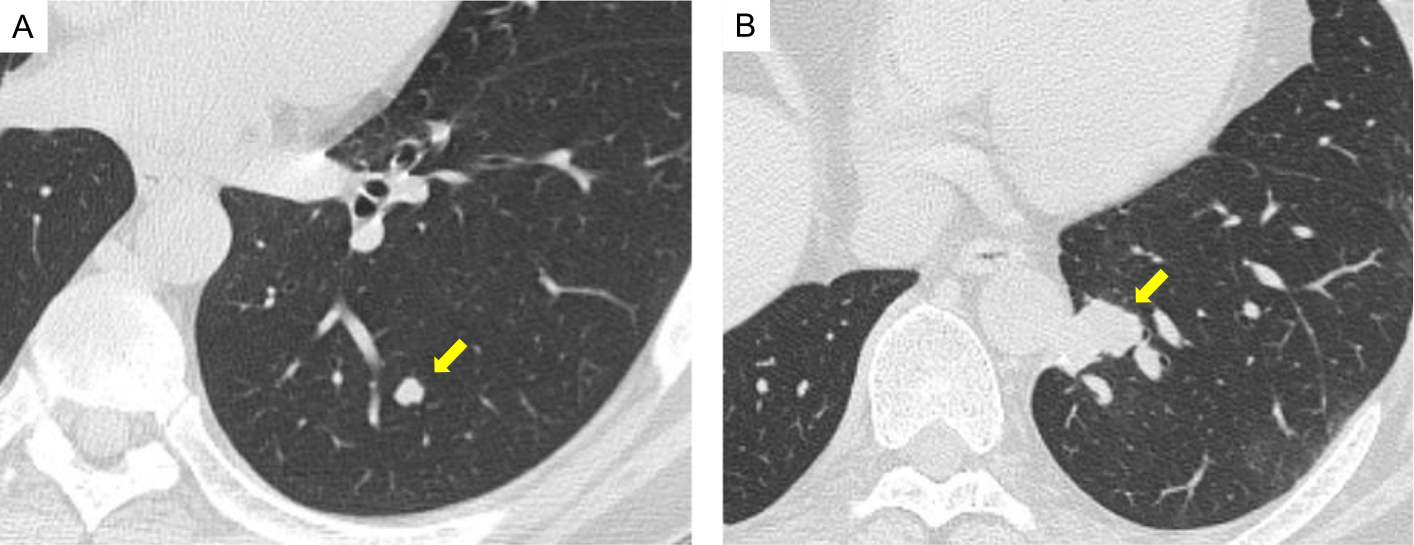
Solitary pulmonary metastasis of synovial sarcoma (SS) in Case 1 (A) and Case 3 (B)

In Case 1, a 63-year-old woman with primary SS in the left ankle underwent pulmonary metastasectomy three times. IHC of SS18-SSX for pulmonary metastases was obtained from the first and second metastasectomy specimens. Figure 1A shows the computed tomographic findings of solitary pulmonary metastasis in the left lower lobe before the initial left wedge resection in this case.

**Fig. 2:**
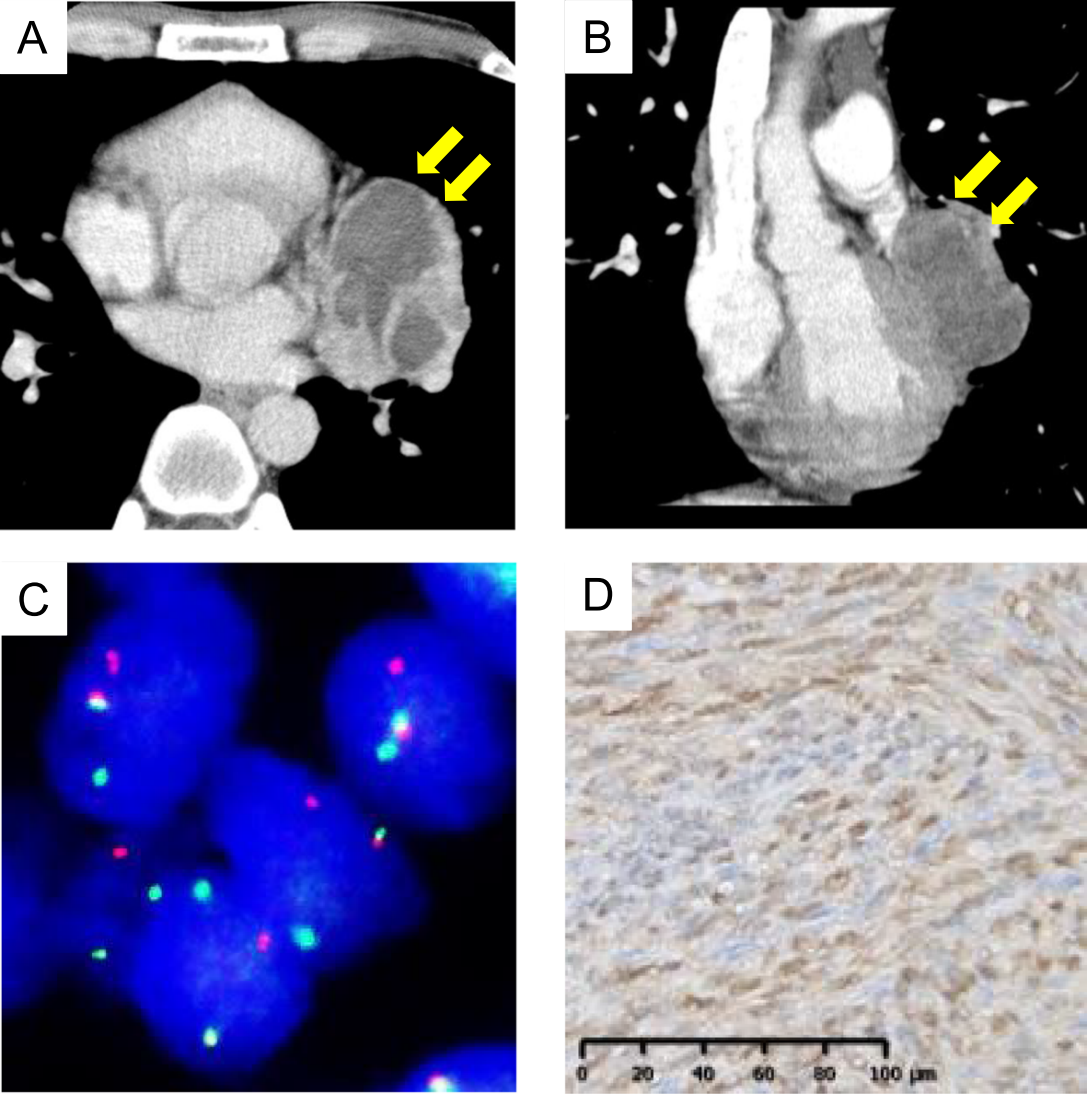
Fluorescence in situ hybridization for SS18 rearrangement and immunostaining of SS18-SSX antibody in Case 2 (A)(B) The chest computed tomography (CT) scan shows a mediastinal mass of size 60 × 60 × 40 mm. (C) Image showing the results of fluorescence in situ hybridization for SS18 rearrangement. A disconnected SS18 gene can be seen. (D) The SS18-SSX fusion-specific antibody (E9X9V) is positive

In Case 2, a 28-year-old man had primary SS in the mediastinum. The large mass (60 × 60 × 40 mm) was protruding from the left side chest cavity (Fig. 2A, B), which was resected with combined pericardial resection by median sternotomy. In this case, the definitive diagnosis of SS was not reached until the initial metastasectomy, which was 108 months after the primary surgery when SS was finally diagnosed through comprehensive pathology workups including SS18 break-apart FISH for primary and metastatic sites. Figure 2C shows FISH for SS18 rearrangement in the primary tissue, demonstrating a disconnected SS18 gene. No evidence of recurrence was observed 17 months after the last pulmonary metastasectomy.

In Case 3, a 33-year-old man with primary SS in the left forearm underwent pulmonary metastasectomy four times and subsequent radiation therapy for right-sided pulmonary metastasis. Figure 1B shows the computed tomographic findings of the solitary pulmonary metastasis in the left lower lobe before left basal segmentectomy. IHC of SS18-SSX for pulmonary metastases was obtained from the first and second metastasectomy specimens.

In Case 4, a 51-year-old man had the primary origin as the left knee. He underwent pulmonary metastasectomy five times. The patient was followed up without any treatment. IHC of SS18-SSX for pulmonary metastases was obtained from the third and fourth metastasectomy specimens.

In Case 5, a 30-year-old man had the primary origin as the right forearm. He underwent pulmonary metastasectomies four times. IHC of SS18-SSX for pulmonary metastases was obtained from the second and third metastasectomy specimens. He had recurrence and was undergoing chemotherapy.

### IHC

**Fig. 3:**
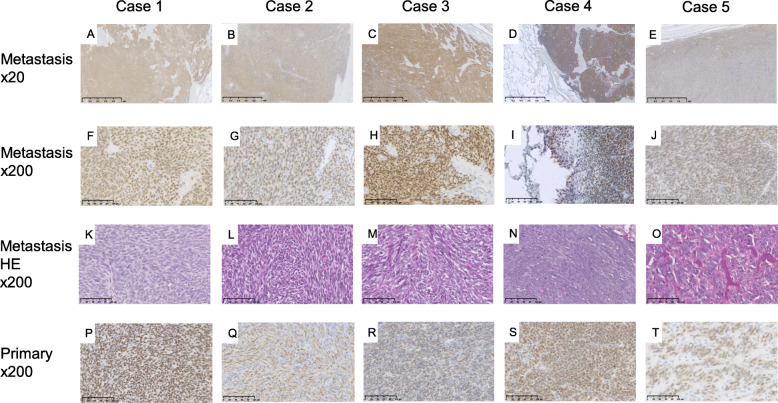
Immunostaining findings of SS18-SSX in the synovial sarcoma with pulmonary metastasis in each case All specimens (A-E: × 20, F-J: × 200) were diffusely positive. Hematoxylin and eosin staining of the synovial sarcoma with pulmonary metastasis (K-O: × 200). Immunostaining findings of SS18-SSX in the synovial sarcoma of primary origin (P-T: × 200). All specimens were positive

**Fig. 4:**
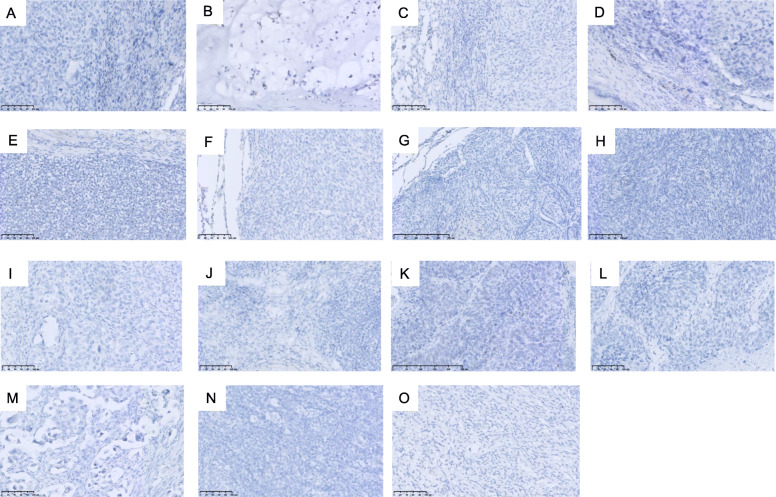
Representative findings of immunostaining of SS18-SSX in other bone soft tissue sarcoma (× 200) (A) Osteosarcoma, (B) Chondrosarcoma, (C) Liposarcoma, (D) Malignant fibrous histiocytoma, (E) Ewing’s sarcoma, (F) Fibrosarcoma, (G) Leiomyosarcoma, (H) Undifferentiated spindle cell sarcoma, (I) Adenocarcinoma, (J) Squamous cell carcinoma, (K) Small cell lung cancer, (L) Large cell lung cancer, (M) Pleomorphic carcinoma, (N) Typical carcinoid, and (O) Solitary fibrous tumor. All cases were negative

Figure 3 shows the histologic findings of SS18-SSX IHC and hematoxylin and eosin of 10 pulmonary metastatic SS and 5 corresponding primary sites from 5 patients with SS. The SS18-SSX fusion-specific antibody was positive with diffusely strong staining in all 10 metastatic SS samples. All five primary SS tumors were stained similarly to the corresponding metastatic SS. However, no staining of SS18-SSX was observed in the 93 clinical and histologic mimics (49 other bone and soft tissue sarcomas, 39 primary lung cancers, and 5 SFTs). Figure 4 shows the histologic findings of SS18-SSX IHC of 15 representative cases from 93 clinical and histological mimics. A summary of the IHC results is presented in Table 2.

**Table 2:** Summary of the results of IHC staining for SS18-SSX antibody

	Number	SS18-SSX-positive ratio
**Synovial sarcoma (metastasis)**	10	100%
**Synovial sarcoma (primary)**	5	100%
**Non-SS pulmonary metastatic tumors**		
Osteosarcoma	8	0%
Chondrosarcoma	7	0%
Liposarcoma	5	0%
Malignant fibrous histiocytoma	9	0%
Ewing’s sarcoma	4	0%
Leiomyosarcoma	9	0%
Fibrosarcoma	3	0%
Undifferentiated pleomorphic sarcoma	4	0%
**Primary lung cancer**		
Adenocarcinoma	9	0%
Squamous cell carcinoma	5	0%
Small cell lung cancer	4	0%
Large cell lung cancer	5	0%
Pleomorphic carcinoma	8	0%
Carcinoid	8	0%
**Solitary fibrous tumor**	5	0%

*IHC* immunohistochemistry, *SS* synovial sarcoma

**Fig. 5:**
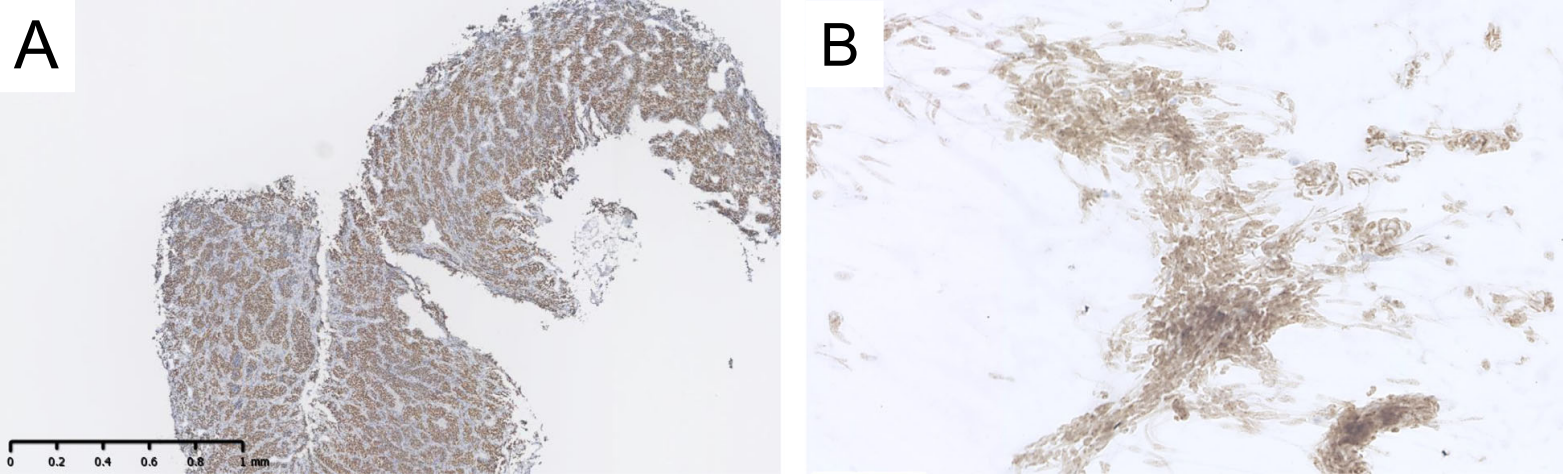
SS18-SSX immunostaining in the synovial sarcoma: Case 5 (A); intraoperative sealed cytology: Case 3 (B). The image shows strong diffuse nuclear staining

Figure 5A shows the histologic finding of SS18-SSX IHC of a percutaneous needle biopsy specimen from the primary right forearm tumor in Case 5, demonstrating diffuse strong staining of SS18-SSX antibody similar to the surgically resected specimen (Fig. 3T). Figure 5B shows the cytological findings of SS18-SSX IHC of intraoperative touch imprint cytology specimen from the pulmonary metastatic site, demonstrating strong staining of the SS18-SSX antibody.

## Discussion

In this study, we demonstrated the usefulness of the IHC of SS18-SSX fusion-specific antibody for the differential diagnosis of pulmonary metastatic SS from clinical and histological mimics. The significant findings of this study are as follows: 1) this is the first study to validate the diagnostic performance of SS18-SSX IHC for the diagnosis of pulmonary metastatic SS; 2) we examined SS18-SSX IHC in clinical and histologic mimics of pulmonary metastatic SS, including various types of primary lung cancers and pulmonary metastatic non-SS sarcomas; 3) the sensitivity and specificity for the diagnosis of pulmonary metastatic SS were high (both 100%); 4) positive SS18-SSX IHC was seen in the biopsy and cytology specimens, suggesting the usefulness of the test in clinical practice; 5) positive SS18-SSX IHC in a rare case of mediastinum-originated SS suggested a potential routine use of the test for the differential diagnosis of intrathoracic indeterminate sarcomas; and 6) the simple and widely prevalent IHC technique for the test with a high diagnostic performance could replace traditional genomic tests such as FISH or RT-PCR.

The standard test for the diagnosis of SS has been genomic tests to detect the SS18-SSX gene fusion by FISH or RT-PCR [13], which have reported to be highly specific tests. However, several studies suggested the relatively low sensitivity (83–94%) of these techniques [16, 17] and their technical and cost-related issues [13]. Alternatively, IHC of transducing-like enhancer split 1 (TLE1) has been recognized to distinguish SS from other soft tissue malignancies [3, 18]. Although TLE1 is reported to show strong and diffuse nuclear staining in SS, the specificity of TLE1 IHC for the diagnosis of SS is low because of its positivity in up to one-third of non-SS sarcomas [3, 15, 19–21]. Recently, Baranov et al. evaluated SSX-SS18 IHC in 100 genetically confirmed primary SS tumors and 300 histologic mimics of SS, demonstrating that SS18-SSX IHC had high sensitivity (95%) and high specificity (100%) for the diagnosis of primary SS and described that the test could replace traditional genomic tests based on its technical simplicity and high diagnostic performance [4]. No study investigated the diagnostic performance of the test for the diagnosis of metastatic SS to date and our current study is the first to validate the usefulness of SS18-SSX IHC for the diagnosis of pulmonary metastatic SS, which demonstrated high sensitivity (100%) and sensitivity (100%) to distinguish SS from clinical and histologic mimics.

Our current study also provides supportive evidence to use SS18-SSX IHC for the differential diagnosis of indeterminate mediastinal sarcomas. Because of the rarity of primary mediastinal sarcomas including SS [22], the routine use of genetic tests for the differential diagnosis of SS would not be realistic in clinical practice. In case 2, the definitive diagnosis of SS was not reached until the initial metastasectomy 108 months after the primary surgery when SS was suspected, and FISH was performed. In such cases, SS18-SSX IHC would be a better alternative option for differentiating SS because of its technical simplicity, relatively low cost, and good diagnostic performance.

More importantly, our current study suggests the potential utility of SS18-SSX IHC for biopsy and/or cytology specimens. In case 5, sufficient staining of SS18-SSX was confirmed by needle biopsy and touch imprint cytology specimens from the primary site of SS. Although SS18-SSX IHC in biopsy or cytology specimens from pulmonary metastatic SS was not assessed in this study, a differential diagnosis of SS could be made using small amounts of tumor tissue or cells obtained by bronchoscopy or imaging-guided biopsy.

The five primary and ten metastatic SS samples in this study were strongly positive for SS18-SSX IHC (100% sensitivity). However, due to uncommon variants of SS18-SSX fusion, the sensitivity of SS18-SSX IHC is not perfect [4, 23]. Tahara et al. suggested RT-PCR and DNA sequencing would be useful to confirm the diagnosis in such cases [23]. Baranov et al., however, suggested that these cases could be recognized by the SSX C-terminus antibody (E5A2C), and therefore, the combination of these IHCs (SS18-SSX and SSX C-terminus) could be the gold standard of molecular genetic or cytogenetic testing in the majority of SS. SSX IHC should be considered in cases with a high suspicion of SS but negative for SS18-SSX.

This study had some limitations. First, the number of SS cases was small, and there were no cases with negative SS18-SSX IHC in the primary site of SS. Therefore, it is unknown whether metastatic sites in such cases could be identified using SS18-SSX IHC. Second, we only tested two selected metastatic sites in each SS case, which might have affected the results of this study. Third, we did not assess SS18-SSX IHC in a primary carcinosarcoma of the lung, which is an important differential diagnosis for SS.

## Conclusions

In conclusion, we showed the usefulness of IHC of SS18-SSX fusion-specific antibody for the differential diagnosis of pulmonary metastatic SS from clinical/histological mimics with high diagnostic performance (100% sensitivity and 100% specificity). We also showed the potential utility of SS18-SSX IHC for the diagnosis of SS arising from rare origins such as the mediastinum and SS obtained by biopsy/cytology specimens. This simple and reliable method could replace traditional genomic tests in terms of the differential diagnosis of pulmonary metastatic SS.

## Data Availability

The data presented in this study can be shared in response to reasonable request to the corresponding author.

## Abbreviations

SS: Synovial sarcoma
FISH: Fluorescence in situ hybridization
RT-PCR: Reverse transcriptase-polymerase chain reaction
IHC: Immunohistochemistry
SFT: Solitary fibrotic tumor
BSA: Bovine serum albumin
PBS: Phosphate-buffered saline
